# Supplementary material for: Safety and biologic activity of a bispecific T cell receptor targeting HIV Gag in males living with HIV: a first-in-human trial
Source: Nat Commun. 2026 Jan 31;17:2207. doi: 10.1038/s41467-026-68833-2 (PMC12963441; doi:10.1038/s41467-026-68833-2)
Supplement: Supplementary file 1 — Supplementary Information [file 41467_2026_68833_MOESM1_ESM.pdf]

Supplementary Information

Title: *Safety and biologic activity of a bispecific T cell receptor targeting HIV Gag in males living with HIV: a first-in-human trial*

Authors: Linos Vandekerckhove, Julie Fox, Borja Mora-Peris, Jordi Navarro, Sabine D. Allard, Alison J. Uriel, Santiago Moreno Guillén, Marta Boffito, Frank Post, Vicente Estrada, Beatriz Mothe, Mareva Delporte, Adel Benlahrech, Haseeb Rahman, James Clubley, Agatha Treveil, Jonathan Chamberlain, Rory Harrison, Miriam Hock, Yuan Yuan, Jason Wustner Sylvie Moureau, Andrew D. Whale, Zoë Wallace, Praveen K. Singh, Kehmia Titanji, Lucy Dorrell, Sarah Fidler

Corresponding author: Linos Vandekerckhove, email: Linos.vandekerckhove@UGent.be

a

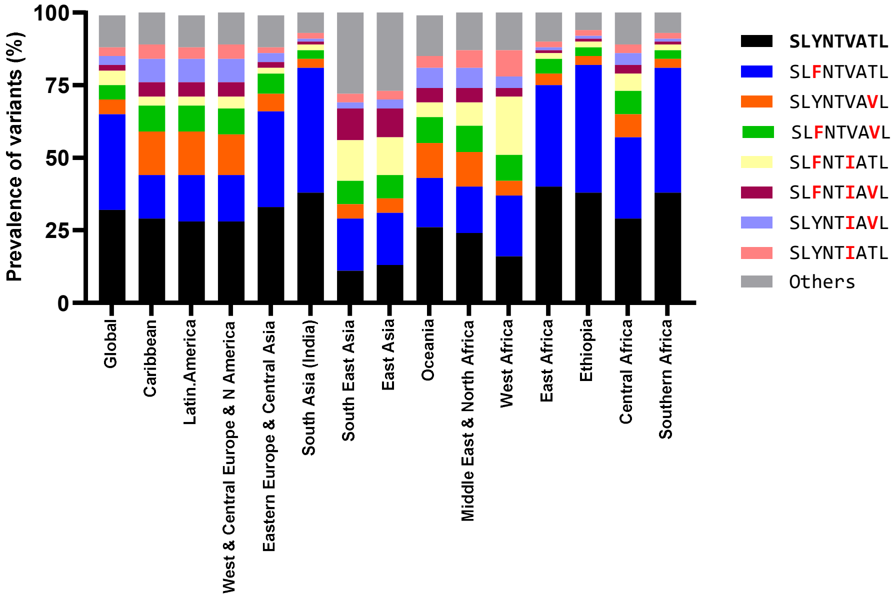

b

Target sequence	$t_{1/2}$ (h)[a]	Binding affinity K_D (nM)	Donor 1 EC_{50} (nM)	Donor 2 EC_{50} (nM)
**SLYNTVATL**	**3.71**	**0.10**	**0.49**	**0.79**
SLYNTIAVL	1.81	0.16	0.37	0.62
SLYNTIATL	3.51	0.20	0.31	0.44
SLFNTIAVL	1.78	0.47	1.22	2.56
SLYNTVAVL	2.03	0.57	0.97	1.09
SLFNTIATL	3.06	0.63	0.57	0.95
SLFNTVATL	3.79	1.31	1.24	1.62
SLFNTVAVL	2.25	1.62	2.87	5.29

Pearson correlation coefficient (K_D vs EC_{50})		
	r	P value
Donor 1	0.8720	0.0048
Donor 2	0.7873	0.0204

c

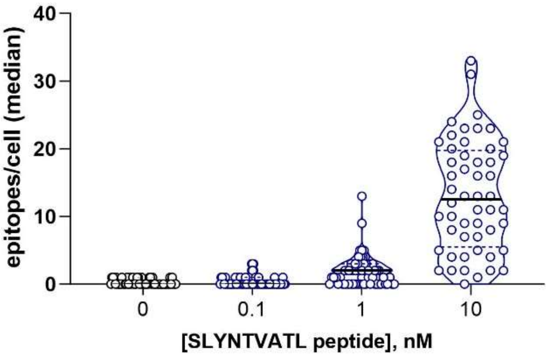

Supplementary Figure 1 | Activity of IMC-M113V against prevalent variants of Gag_{77-85} in vitro

a. Bar graphs showing worldwide relative prevalence of Gag_{77-85} variants present at frequencies >2% in the Los Alamos National Laboratory HIV sequence database. Variants present at frequencies <2% and all subvariants are grouped and shaded in grey.

b. Stability ($t_{1/2}$) of the 8 most prevalent Gag_{77-85} variants in complex with HLA-A*02:01 and IMC-M113V binding affinities (25 °C) to these variants presented as K_D values. EC_{50} values are derived from a T cell redirection (IFN-γ ELISpot) assay in which PBMC from two HIV-naïve donors were cultured with T2 cells pulsed with increasing concentrations of variant peptides in the presence of IMC-M113V (100 pM). The K_D and EC_{50} values are also denoted by shaded bars within the columns. Pearson correlation coefficient was used to evaluate the relationship between binding affinities (K_D) and in vitro potency (EC_{50}) of IMC-M113V against the variants (right-hand table).

c. T2 cells pulsed with a titration of Gag_{75-85} peptide (0.1 to 10 nM) or left unpulsed were stained with the rabbit Fc-tagged TCR domain of IMC-M113V^{RES} and goat anti-rabbit CF640R. TCRs bound to peptide-HLA complexes on cells were quantified by total internal reflection microscopy (bottom panel). Each dot represents one cell. Horizontal solid bars indicate median values and dotted lines indicate upper and lower quartiles.

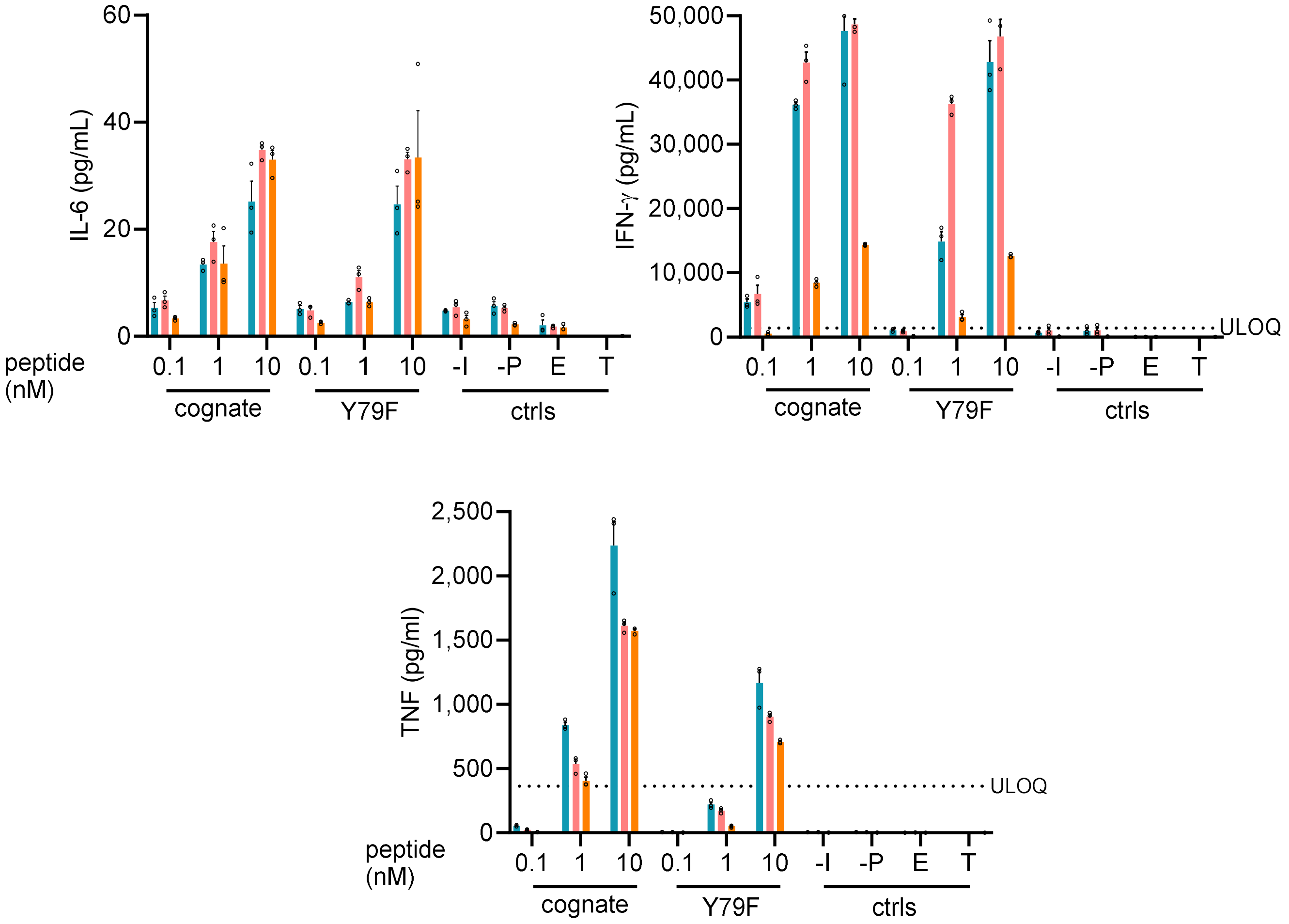

Supplementary Figure 2 | IMC-M113V^{RES}-induced cytokine response is dependent on affinity for target peptide and peptide concentration

PBMC (E) from HIV-negative donors were cultured with Gag$_{77\text{-}85}$ (cognate or major variant sequence) peptide-pulsed T2 cells (T) at a ratio of 10:1 and IMC-M113V^{RES} (I) overnight following which cytokine production (IL-6, IFN-γ and TNF) was quantified by MSD immunoassay. Mean ± SEM of triplicates are presented above. No cytokine induction above background levels was observed in the control conditions (ctrls): absence of ImmTAV (-I), peptide (-P), E alone or T alone. Upper limit of quantification (ULOQ) is indicated by the dotted lines.

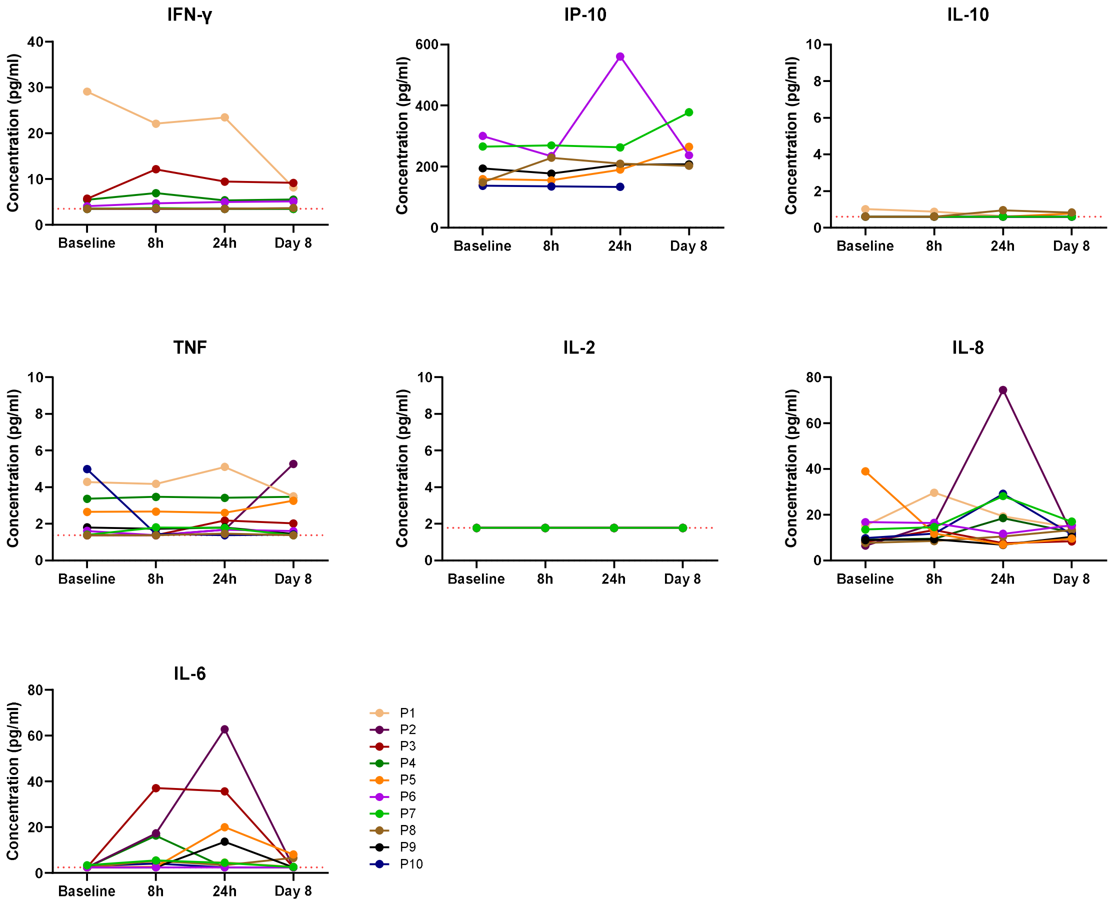

Supplementary Figure 3 | Quantification of serum cytokines pre and post a single 15 µg dose of IMC-M113V

Serum cytokines were quantified in samples obtained pre-infusion (baseline), 8 and 24 hours and 7 days post-infusion. Absolute values for participants in Cohort 3 (15 µg) are presented. Each coloured line represents an individual study participant. Dotted line indicates lower limit of quantification (LLOQ) for each cytokine.

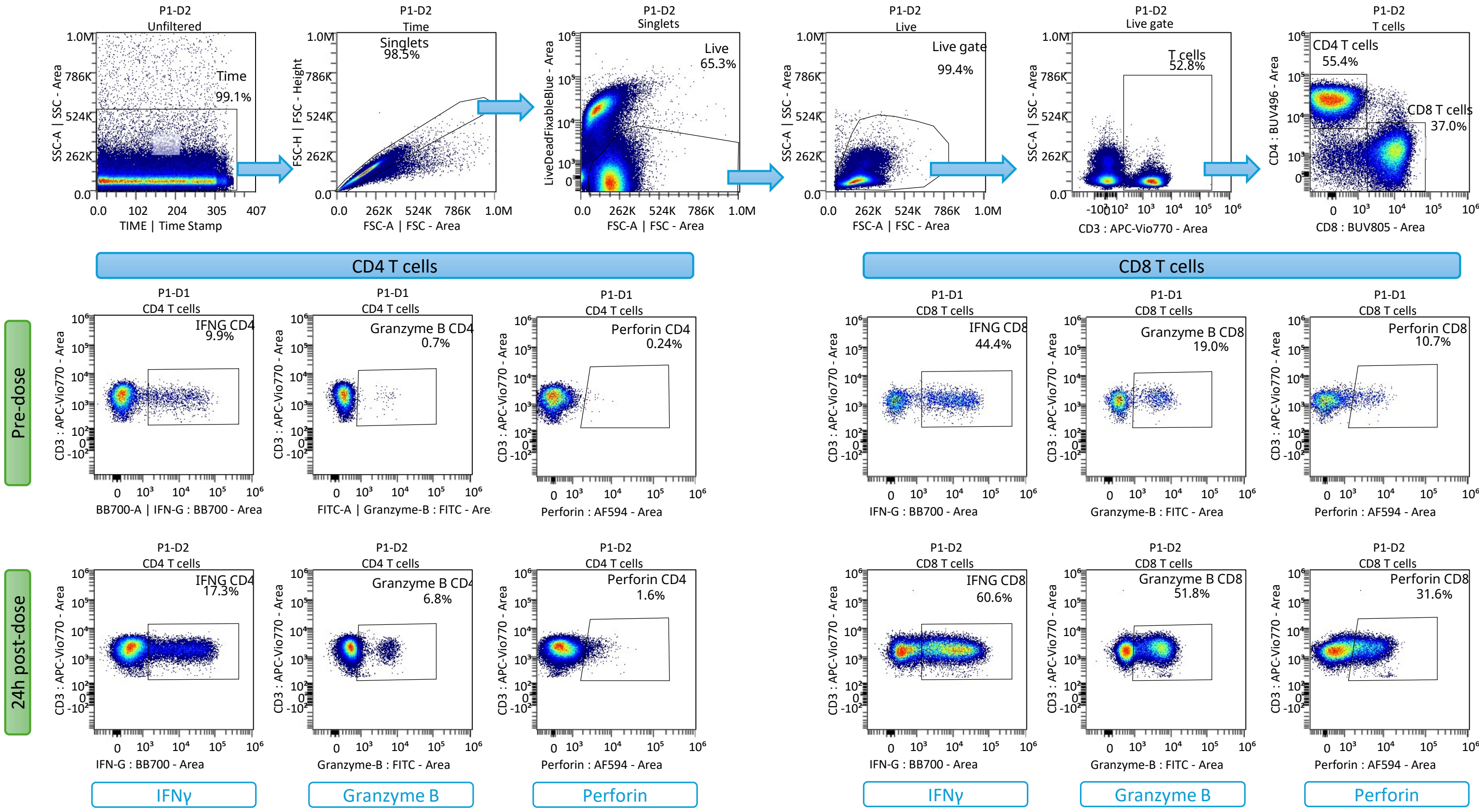

Supplementary Figure 4 | Flow cytometry gating strategy for T cell cytolytic markers

CD4+ and CD8+ T cells were identified within CD3+ live (top row). IFN-γ, granzyme B, and perforin expression were determined based on fluorescence minus one and unstimulated controls. Representative dot plots are shown for pre-dosing (middle row) and 24h post-dosing (bottom row) samples. This gating strategy was used to generate IFNγ, granzyme B, and perforin data shown in Fig 5d.

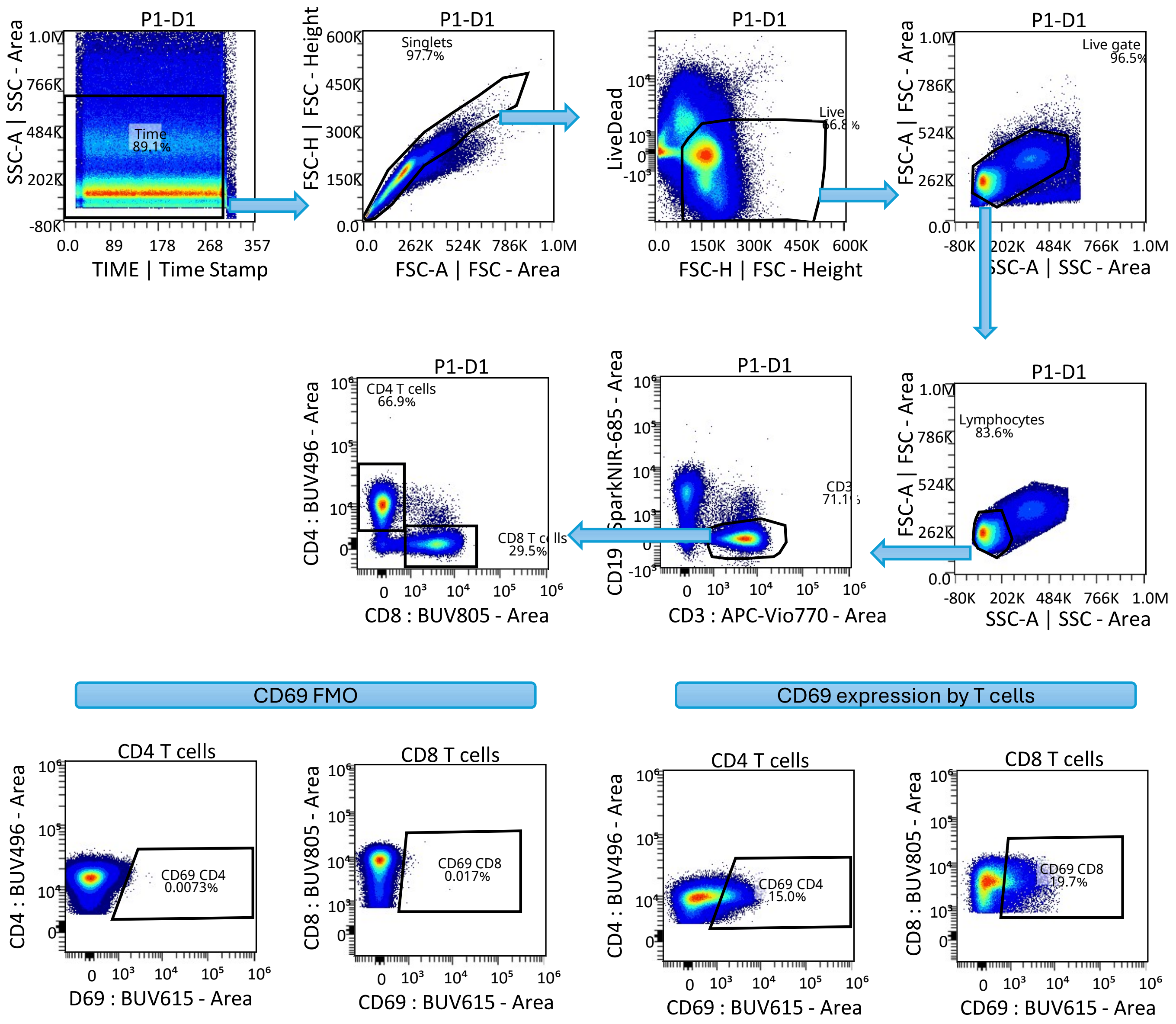

Supplementary Figure 5 | Flow cytometry gating strategy for T cell activation

CD4 and CD8 T cells were identified within CD3+ live cells (top and middle rows). CD69 expression by CD4 and CD8 T cells (bottom right panel) was determined based on fluorescence mine one controls (bottom left panel). This gating strategy was used to generate CD69 data shown in Fig 5d.

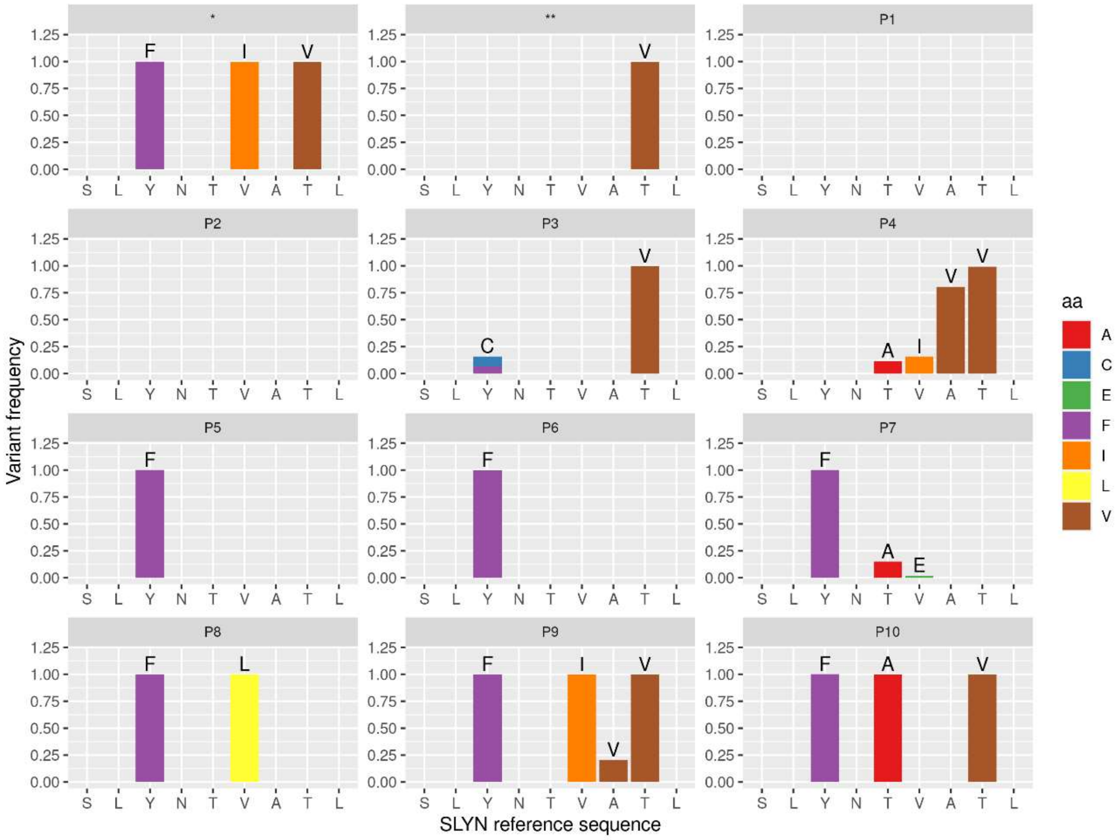

Supplementary Figure 6 | Baseline cell-associated HIV RNA-derived Gag$_{77-85}$ sequences

Gag$_{77-85}$ sequences were determined in the participants at baseline from cell-associated RNA in ex vivo CD4+ cells using Next-Generation Sequencing (Illumina MiSeq). Intra-patient Gag$_{77-85}$ variants (relative to the cognate "SLYNTVATL" sequence) detected at a frequency of >3% in the sequencing reads are presented; (*1.6 µg; **5 µg; P1-10 = 10 participants in the 15 µg cohort).

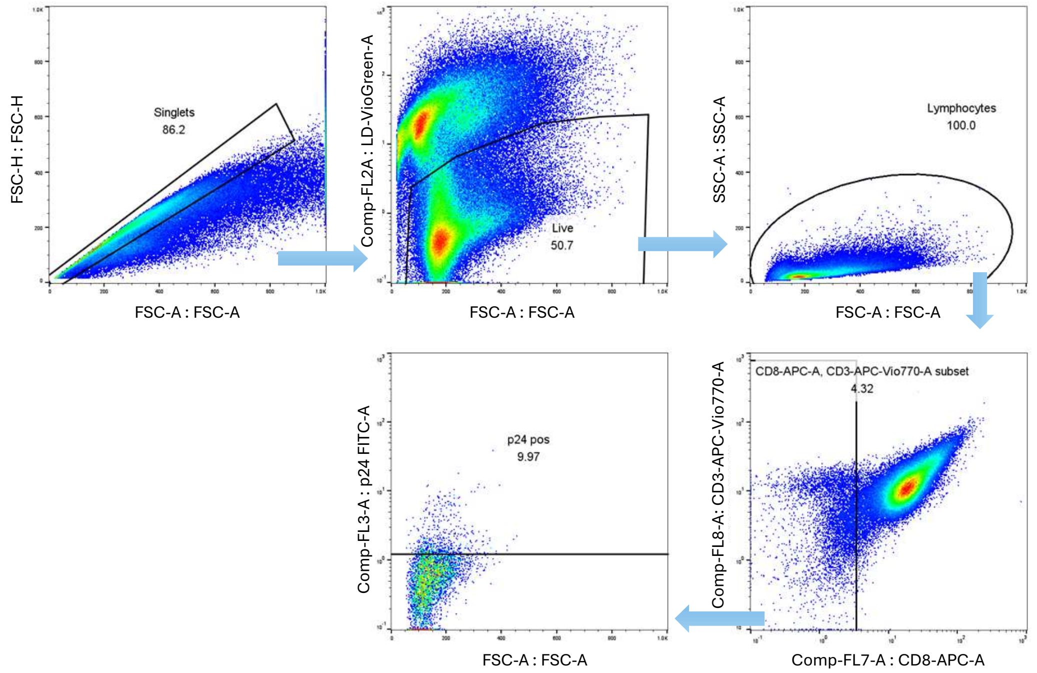

Supplementary Figure 7 | Representative flow cytometry gating for intracellular Gag p24 staining

HLA-A*02:01-transduced C8166 cells were infected with HIV-1 IIIB or mutated pNL4-3 then co-cultured with or without CD8+ T cells together with IMC-M113V. Live single C8166 cells were identified (top row) followed by exclusion of CD8 T cells and identification of infected cells based on intracellular Gag p24+ staining (bottom row). This gating strategy was used to generate data shown in Fig 1c, 2a, and 2b.

Supplementary Table 1. Adverse events

**Adverse Event**	**All Grades**	**Grade 1**	**Grade 2**
Possibly related			
Fatigue	2	1	1
Skin redness	1	1	
Eczema (Face)	1	1	
Eczema (Gluteus)	1		1
Herpes Simplex Virus	1	1	
Itch (Gluteus)	1	1	
Phlebitis	1	1	
Not related			
Burn wound on hand	1		1
Common cold	1		1
Decreased WBC	1	1	
Symptoms of upper respiratory tract infection (cough, clear sputum)	1	1	
Sprained ankle	1	1	
Upper respiratory tract infection	1	1	
SARS-CoV-2 infection	1	1	
Total	15	11	4

Supplementary Table 2. Antibodies used in the study

Marker	Fluorochrome	Clone	Cat #	Supplier	Dilution
Quantification of pHLA complexes on Gag_{77-85} positive cells					
Anti-Rabbit IgG	CF640R	-	BT20176-1	Biotium	1:2000
Annexin V	Alexa Fluor 488	-	A13201	Thermo Fisher	1:20
HIV Gag p24 intracellular staining					
CD3	APC*Cy7	HIT3a	300318	BioLegend	1:100
CD8	APC	SK1	344722	BioLegend	1:100
P24	FITC	KC57	6604665	Beckman Coulter	1:50
T cell activation and effector function					
CCR7	BUV395	2-L1-A	749655	BD Biosciences	1:25
CD154 (CD40L)	BV480	TRAP1	746337	BD Biosciences	1:25
CD19	SPARK NIR 685	HIB19	302270	BioLegend	1:50
CD25	BUV563	2A3	612918	BD Biosciences	1:200
CD28	BUV615	L293	751270	BD Biosciences	1:50
CD3	APC-Vio770	REA613	130-113-136	Miltenyi	1:50
CD39	BUV661	TU66	749967	BD Biosciences	1:200
CD4	BUV496	SK3	612936	BD Biosciences	1:25
CD45RO	BV570	UCHL1	304226	BioLegend	1:50
CD69	BV711	FN50	563836	BD Biosciences	1:100
CD8	BUV805	SK1	612889	BD Biosciences	1:25
CD95	BUV737	DX2	612790	BD Biosciences	1:25
FoxP3	PE.Cy5.5	PCH101	35-4776-42	Thermo Fisher Scientific	1:100
Granzyme B	FITC	GB11	560211	BD Biosciences	1:10
IFNg	BB700	B27	566394	BD Biosciences	1:50
IL-13	BB660	JES10-5A2	Custom	BD Biosciences	1:25
IL17A	BV605	BL168	512326	BioLegend	1:50
IL-2	BV421	MQ1-17H12	564164	BD Biosciences	1:25
IL-4	BB660	8D4-8	Custom	BD Biosciences	1:100
IL-6	BB630	MQ2-13A5	Custom	BD Biosciences	1:50
Ki-67	BV650	B56	563757	BD Biosciences	1:100
Perforin	AX594	B-D48	NBP3-14581AF594	Novus Biologics	1:200
TNF	BV750	Mab11	566359	BD Biosciences	1:25
Viability	Live Dead UV Blue	-	L23105	Thermo Fisher Scientific	1:1000

Supplementary Table 3. Primers and probes used for quantification of HIV *gag* CA-RNA

ID	*gag*_Primer_F (5'-3')	*gag*_Primer_R (5'-3')	*gag*_Probe (5'-3')
**P1, 3, 5-6, 8-10**	GACTAGCGGAGGCTAG AAGGAGAG	CTAATTTTCCSCCDC TTAATAYTGACG	AT+G+GGT+GC+GAGA
**P4**	GACTAGCGGAGGCTAG AAGGAGAG	CTAATTTTCCSCCDG ATAATAYTGACG	AT+G+GGT+GC+GAGA
**P2, 7**	AGTTGGAGGACATCAA GCAGCCATGCAAAT	TGCTATGTCAGTTCC CCTTGGTTCTCT	GACCATCAATGAGGAAG CTGCAGAATGGGAT

1. PROTOCOL SUMMARY

1.1. Synopsis

Protocol Title

An open-label dose-escalation study evaluating the safety, pharmacokinetics (PK) and antiviral activity of IMC-M113V in HLA-A*02:01-positive subjects with chronic human immunodeficiency virus (HIV) infection who are virologically suppressed.

Short Title

Phase 1/2 study of IMC-M113V in virologically suppressed chronic HIV infection

Rationale

IMC-M113V is an immune-mobilizing monoclonal T cell receptor against viruses (ImmTAV®), a new class of bispecific protein therapeutic designed to deliver targeted elimination of HIV reservoirs in individuals with chronic HIV infection. IMC-M113V comprises a soluble, affinity-enhanced T cell receptor (TCR; targeting domain) fused to an antibody single-chain fragment variable that specifically recognizes cluster of differentiation 3 (anti-CD3 scFv; effector domain). The IMC-M113V TCR recognizes a complex consisting of a peptide derived from the HIV Gag matrix protein p17, presented by a human leukocyte antigen, HLA-A*02:01, on the surface of infected CD4+ cells. Once the soluble TCR is engaged, the scFv effector domain can bind to CD3 on any T cell in the vicinity, stimulating the T cell to release effector cytokines and to lyse the bound target cell.

Antiretroviral therapy (ART) is effective in suppressing HIV replication but is incapable of eliminating the virus because it is able to integrate permanently into host chromosomes, resulting in the formation of a cellular reservoir within the first few days of infection. Replication-competent viral genomes may persist in long-lived CD4+ T cells and re-fuel virus production if ART is interrupted, necessitating lifelong administration of ART to prevent relapse. Finite interventions that can eradicate viral reservoirs or reduce them to low enough levels to enable long-term drug-free control (“functional cure”) are therefore highly desirable.

HIV reservoirs have proven to be refractory to immunotherapeutic approaches to date because of low antigen expression, pre-existing viral mutational escape and sequestration in immune-privileged sites. Furthermore, HIV-specific cytolytic T cells (CTLs) exhibit persistent functional deficits even after long-term ART.

Preclinical studies have shown that IMC-M113V can eliminate HIV-infected CD4+ T cells through redirection of polyclonal (non-HIV-specific) CD8+ T cells, bypassing HIV-specific immune effectors that have become dysfunctional. Moreover, IMC-M113V is sensitive to extremely low levels of viral antigen and is thus able to trigger cytolytic activity against HIV-infected cells that evade detection by natural CTLs.

IMC-M113V-103 is a first-in-human (FIH) study designed to assess the safety, tolerability, and pharmacokinetic (PK) profile of IMC-M113V in single and multiple dose regimens, and to explore the effects of these regimens on HIV reservoirs in HLA-A*02:01-positive people living with HIV (PLWH) who are receiving effective ART. The aim of this study is to identify safe, tolerable, and pharmacologically active dosing regimens of IMC-M113V for further clinical development.

Objectives and Endpoints

Objectives	Endpoints
**Primary**	
**Part 1: Single Ascending Dose (SAD) Study**<br>To evaluate the safety and tolerability of IMC-M113V when administered as a single dose during ART<br>**Part 2: Multiple Ascending Dose (MAD) Study**<br>To evaluate the safety and tolerability of IMC-M113V when administered in a multiple dose schedule, up to at least week [redacted], in participants receiving ART	• Incidence and severity of treatment-emergent adverse events (TEAEs)<br>• Incidence of dose-limiting toxicities (DLTs)<br>• Changes in safety laboratory parameters, vital signs, and electrocardiogram (QTcF)<br>• Incidence of serious adverse events (SAEs) and AEs leading to treatment interruption, dose reduction, or discontinuation through 28 days after the last infusion of study treatment
**Secondary**	
To characterize the PK profile of IMC-M113V in single dose and multiple dose schedules	IMC-M113V PK parameters (e.g., AUC, C_{max}, T_{max}, $t_{1/2}$) at multiple time points from baseline up to 72 hours post-dose in SAD and MAD (first dose) and after each subsequent dose in MAD studies
To evaluate incidence of anti-IMC-M113V antibody formation following single and multiple infusions	Incidence of anti-IMC-M113V antibody formation following administration of one or more doses of study drug
To determine pharmacodynamic (PD) changes in the systemic immune response in relation to treatment with IMC-M113V, including but not limited to changes in peripheral cytokines and lymphocyte counts	Change in serum cytokines/chemokines and peripheral blood lymphocyte counts (absolute values and fold-change) from baseline through 72 hours post-dosing with IMC-M113V in SAD and MAD schedules and during Follow-Up
To determine the incidence and duration of post-treatment control during analytical therapy interruption in participants completing multiple dose schedules	• Proportion of participants with pVL < 200 copies/mL 12 weeks after interruption of ART (W24)<br>• Proportion of participants resuming ART before W24<br>• Duration of post-treatment control (pVL < 200 copies/mL) after interruption of ART<br>• Duration of virological suppression (pVL <[redacted] copies/mL after interruption of ART
To determine the recommended Phase 2 dosing regimen	Identification of at least 1 tolerable dosing regimen for further evaluation in subsequent development

Objectives	Endpoints
**Tertiary/Exploratory**	
To assess the antiviral effects of IMC-M113V following administration of SAD and MAD schedules using HIV reservoir measurements, including but not limited to, ex vivo and inducible multiply-spliced HIV RNA, ex vivo and inducible Gag p24 expression, total and integrated HIV DNA and intact and defective proviruses	Incidence of virologically suppressed participants with decrease (expressed as percentage or fold change or log_{10} change in viral parameter [per million CD4+ T cells or PBMC]) from baseline through end of treatment (EOT)
To determine potential PD changes in the systemic immune response following treatment with IMC-M113V	Changes in circulating T cell phenotype, including but not limited to, T cells expressing activation and exhaustion markers (percentage of parental populations) from baseline through EOT
To determine impact on HIV-specific T cell responses in blood	Frequency of HIV-specific T cells (percentage of parental populations) from baseline through EOT
To determine the presence of the IMC-M113V target index sequence, or variants thereof, in blood as a predictor of clinical response	Prevalence of IMC-M113V target index sequence and variants detected in blood prior to, and after receipt of study drug

Overall Design

IMC-M113V-103 is a multi-center open-label dose-escalation Phase 1/2 FIH study in HLA-A*02:01-positive adults with chronic HIV infection who are virologically suppressed on ART. It will evaluate the safety, tolerability, PK and anti-HIV activity of single and multiple doses of IMC-M113V. The study will be conducted in two parts: Part 1 will evaluate single ascending doses and Part 2 will evaluate multiple ascending dose schedules.

Disclosure Statement

This is an open-label, sequential treatment study with two arms with no masking.

Number of Participants

Approximately 26-106 participants are expected to be enrolled and treated in the study, including approximately 14-26 participants in Part 1: SAD (assuming 2 single participant cohorts and 3-5 cohorts of 4 participants [i.e., 5-7 cohorts in total] and allowing for an additional 4 participants in the case of DLTs) and approximately 12-80 participants in Part 2: MAD (assuming approximately 3-8 cohorts of 4 participants and expansion of at least 3 cohorts to up to 20 participants).

Intervention Groups and Duration

Part 1 will be approximately [redacted] weeks for each participant, comprising a maximum [redacted]-day Screening period, a [redacted]-day treatment period involving a single administration of IMC-M113V and a [redacted]-week Follow-Up period, for a total of [redacted] visits. Visits will take place on Days ≤[redacted], [redacted], [redacted], [redacted], [redacted], [redacted], and [redacted]

Part 2 will be a maximum of [redacted] weeks for each participant, comprising a maximum [redacted]-day Screening period, a [redacted]-week treatment period, an analytical therapy interruption (ATI) period of up to [redacted] weeks, and a [redacted]-week Follow-Up (FU) period. The treatment period will involve [redacted] administration of IMC-M113V concurrent with the participant's ART regimen. IMC-M113V will not be administered during the ATI period. Instead, participants will temporarily discontinue ART and pVL will be monitored every [redacted]-[redacted] weeks. Participants will resume ART if they meet pre-specified criteria. The FU period will begin with resumption of ART; re-suppression of pVL will be confirmed by Week (W) [redacted]. There will be up to [redacted] visits, comprising the Screening visit, 12 visits during the treatment period, [redacted]-[redacted] visits during the ATI phase (up to end of W[redacted]), and [redacted] FU visits at W[redacted] and W[redacted] post-ART re-start.

If emerging safety, PK, and PD data obtained in Part 1 or Part 2 support a less frequent dosing schedule relative to [redacted] administration (e.g., every [redacted] week), additional cohort(s) may be opened in Part 2 to explore this (see Section 4.1.2 and Section 10.8).

Administration of Study Intervention

IMC-M113V will be administered by intravenous (IV) infusion over [redacted] hour, under medical supervision in a facility with immediate access to medications and resuscitation equipment to manage cytokine release syndrome (CRS) and other potential acute infusion-related emergencies.

1. Participants will receive premedication consisting of a non-steroidal anti-inflammatory agent (paracetamol 1 g oral or equivalent or ibuprofen 600-800 mg or equivalent) and antihistamine (cetirizine 10 mg oral or equivalent) within 30 minutes of planned start of IMC-M113V infusion.
2. Corticosteroids (prednisolone 30 mg orally or equivalent) may be added to the premedication regimen for subsequent participants at any dose level (and subsequent higher dose levels) where any of the following are observed:
    a. A single Grade 2 CRS episode,
    b. Two or more Grade 1 CRS episodes that do not resolve within 6 hours of onset despite medical intervention, or
    c. Any Grade 1 CRS episode, which in the opinion of the Investigators or the Sponsor, may increase the risk of observing ≥ Grade 2 CRS at the current dose level.
3. Participants will be observed for, at minimum, [redacted] hours after the end of IMC-M113V infusion in Part 1 and following the first [redacted] doses in Part 2. The extended monitoring period may be lengthened to enable further monitoring, if clinically indicated. The extended monitoring may be discontinued for a selected dose that has been deemed to be safe and not associated with CRS of Grade ≥ 2 or any other AE of Grade ≥ 3 in ≥ 10 participants during Part 1 or Part 2 (see Section 6.5.1 and Section 6.5.3). Subsequent participants receiving this dose and not undergoing extended monitoring will undergo additional safeguards as outlined in Section 6.1.1.

Data Monitoring Committee

A Study Safety Team (SST) comprising, at minimum, the Investigators, Sponsor, and Medical Monitor will be responsible for overseeing the safety of the study participants and managing the cohort dose escalation decisions.

An independent Data Monitoring Committee (DMC) comprising at least 3 members will be convened. The remit of the DMC will be outlined in a pre-specified charter and will include evaluation of PD biomarker data during Part 1 and pVL data during the ATI phase of Part 2. At a minimum, the DMC will convene at the following times:

- When safety data from the Day [redacted] visit are available for all the cohorts in Part 1.
- After completion of Part 2.

Additional ad-hoc DMC meetings may be scheduled on request from the Sponsor or the DMC.

5. STUDY POPULATION

Prospective approval of protocol deviations to recruitment and enrolment criteria, also known as protocol waivers or exemptions, is not permitted.

5.1. Inclusion Criteria

Participants are eligible to be included in the study only if all of the following criteria apply:

Age

1. 18-65 years inclusive, at time of informed consent

HLA

2. HLA-A*02:01-positive (central laboratory testing)

Weight

3. ≥ 50 kg

Condition Under Study and Prior Therapy

4. Documented evidence of HIV-1 infection

5.1 On continuous ART for a minimum of 12 months and maximum of 15 years at the time of planned first dose

NOTE*: Must be currently on a stable regimen without plans to modify ART during the study period.*

6. Plasma HIV RNA < 50 copies/mL at Screening and at all available determinations in the 12-month period preceding Screening

NOTE*: Isolated single values ≥ 50 and < 200 copies/mL will be allowed if subsequent determination is below 50 copies/mL.*

7.1 Current CD4+ T cell count > 450 cells/µL and CD4+ cells > 15% total lymphocytes

8. CD4+ T cell nadir > 200 cells/µL

Contraception

9. Participants who engage in sexual activity which could result in pregnancy for themselves or their partner(s) must agree to use highly effective methods of contraception from the trial Screening date until 3 months after the final dose of the study intervention or longer if required by local regulations; cessation of contraception after this point should be discussed with a responsible physician. Highly effective methods of contraception are described in Section 10.4.
10. Participants are not allowed to donate sperm from the time of enrolment until 3 months post-administration of study interventions or longer if required by local regulations.
11. Participants must refrain from egg donation during the study.

Informed Consent

12. Capable of giving signed informed consent as described in Section 10.1, which includes compliance with the requirements and restrictions listed in the informed consent form (ICF) and this protocol.

5.2. Exclusion Criteria

Participants are excluded from the study if any of the following criteria apply:

Condition Under Study and Prior/Current Antiretroviral Therapy

1. Known HIV controller: pVL < 2000 copies/mL in absence of ART for at least 12 months and on ≥ 2 determinations.
2. Initiated ART within 12 weeks of a diagnosis of primary HIV infection, confirmed by any of the following:
    a. Positive HIV-1 serology ≤ 12 weeks after a documented negative HIV-1 antibody (Ab) test,
    b. Negative HIV Ab test plus either positive p24 antigen (Ag) test or detectable HIV RNA,
    c. HIV-1 Ab avidity test consistent with recent infection, or
    d. Weakly reactive or equivocal 4th generation HIV Ab/Ag test.
3. Prior AIDS-defining condition will preclude participation in Part 1 if diagnosed within 90 days prior to Screening. A prior AIDS-defining condition at any time will preclude participation in Part 2.
4. Individuals receiving an ART regimen containing a non-nucleoside reverse transcriptase inhibitor may not enroll in Part 2 unless willing and able to switch to a short-acting alternative prior to receiving their first dose of study drug.

    NOTE: *Individuals receiving a long-acting agent that may be active after ART interruption may not enroll in Part 2.*

Medical Conditions

5. Co-infection with HBV (defined as positive HBsAg test or detectable HBV DNA) or HCV infection (defined as detectable HCV RNA).

    NOTE: *Individuals who have documented spontaneous clearance or curative treatment of HCV are not excluded.*
6. Current active *Mycobacterium tuberculosis* infection or known untreated latent infection.
7. History of clinically significant cardiovascular disease or impaired cardiac function, including any of the following:
    a. Congestive heart failure (New York Heart Association Class ≥ 3).
    b. Uncontrolled hypertension (consistent findings of systolic blood pressure [BP] > 160 mmHg or diastolic BP > 110 mmHg, as defined in Section 8.5.2).
    c. History of ventricular arrhythmia currently requiring medical treatment or uncontrolled atrial fibrillation.
    d. QTcF > 470 msec on Screening electrocardiograms (ECGs) or known history of congenital prolonged QT syndrome.
    e. Acute myocardial infarction or unstable angina pectoris ≤ 6 months prior to Screening.

8. Active autoimmune disease requiring immunosuppressive treatment, including inflammatory bowel disease (ulcerative colitis or Crohn's disease), within 2 years of Screening.

   ***NOTE**: Participants with vitiligo, alopecia, managed hypothyroidism (on stable replacement doses), asymptomatic adrenal insufficiency (on stable replacement doses), psoriasis, resolved childhood asthma/atopy, well-controlled asthma, and Type I diabetes mellitus (HbA1c < 7%) are not excluded.*

9. Participants with prior solid organ or bone marrow transplant.

10. History of malignant disease will preclude participation if diagnosed in the preceding 2 years from the planned first dose of IMC-M113V; in addition, history of systemic virus-associated cancer, including Kaposi's sarcoma and lymphoma, will preclude participation in Part 2 if diagnosed at any time.

    ***NOTE**: The following exceptions are permitted:*

    - *Malignancies other than those specified above that were treated curatively and have not recurred within 2 years from the planned first dose of IMC-M113V.*
    - *Completely resected basal cell and squamous cell skin cancers.*
    - *Any malignancy considered to be indolent and that has never required therapy, with exception of indolent lymphomas, such as chronic lymphocytic leukemia, which are still exclusionary.*
    - *Completely resected carcinoma in situ of any type.*

11. Pregnant or lactating women.

Prior/Concomitant Therapy

12. Use of blood products, cytokine therapy or other immunotherapy or immunosuppressive medication in the preceding 3 months from Screening.

13. Current or recent systemic steroid therapy (in the preceding 3 months from Screening) or anticipated need for systemic steroids during the study with the following exceptions:

    a. Treatment for well-controlled and asymptomatic adrenal insufficiency is permitted, but replacement dosing is limited to prednisone ≤ 12 mg daily or the equivalent.
    b. Local steroid therapies (e.g., optic, ophthalmic, intra-articular, or inhaled medications) are acceptable.
    c. Premedication for allergy to contrast reagent.

Prior/Concurrent Clinical Study Experience

14. History of any investigational HIV immunotherapy or vaccine within 6 months of Screening.

15. Planned receipt of vaccines: live vaccines are not permitted within 28 days, and non-live vaccines within 14 days of planned first administration of IMC-M113V.

16. Participants must not have received prior treatment with an ImmTAC, including tebentafusp, IMC-C103C or IMC-F106C,

17. Participation in other interventional studies is not permitted during the treatment and ATI periods of this study. Participation in observational studies may be permitted, after consultation with, and approval by the Medical Monitor.

Diagnostic Assessments

18. If any of the following laboratory exclusion criteria are met, then the site may have the participant retested. If a single value is within ± 10% of the listed laboratory exclusion criterion value upon retest, and the value is considered not clinically significant by the physician Investigator, the participant may be considered for enrolment:
    a. Hemoglobin < 120 g/L for participants assigned male at birth; < 110 g/L for participants assigned female at birth
    b. Platelet count < 150 × 10^9/L
    c. Alanine aminotransferase (ALT) > 3 × ULN (upper limit of normal)
    d. eGFR (Foundation 2009) < 60 mL/min/1.73 m^2 (calculated using CKD-EPI equation, 2009; or measured)

Other Exclusions

19. For subjects enrolling in Part 2, inability or unwillingness to adhere to safer sex practices during ART interruption.
20. Known or suspected hypersensitivity or previous severe reactions to any of the constituents of IMC-M113V, or the drugs used in the pre-medication regimen (e.g., corticosteroids, anti-histamines, non-steroidal anti-inflammatory agents, paracetamol)
21. Any medical condition that would, in the Investigator's judgement, interfere with the patient's participation in the study due to safety concerns, compliance with study procedures or interpretation of study results.
22. Compromised lung function resulting in persistently reduced oxygen saturation on air (Sp02 or Sa02) < 93%. NOTE: Temporary hypoxia associated with acute respiratory disease is not exclusionary if fully resolved at the time of Screening.

5.3. Lifestyle Considerations

Participants must be willing to adhere to the following restrictions during the study:

- Refrain from alcohol consumption and/or drug substance use that is likely to prevent adequate adherence to study procedures.
- Refrain from unaccustomed physical activity from the time of Screening to last study-related visit.
- Adhere to safer sex practices during the ATI (Section 10.4).

5.4. Screen Failures

Screen failures are defined as participants who consent to participate in the clinical study but failed to start treatment for any reason. A minimal set of screen failure information is required to ensure transparent reporting of screen failure participants to meet the Consolidated Standards of Reporting Trials (CONSORT) publishing requirements and to respond to queries from regulatory authorities. Minimal information includes demography, screen failure details, eligibility criteria, and any serious adverse event (SAE). The date of consent for Screening will also be collected.

Individuals who do not meet the criteria for participation in this study (screen failure) may generally be rescreened after abnormal laboratory results, test results, or symptoms are subsequently corrected or treated, but these shall first be discussed and agreed upon with the

Sponsor Medical Monitor. Upon repeat Screening, only those labs or tests that were abnormal need to be repeated if they are within the appropriate Screening window. Rescreened participants shall be assigned the same participant number as for the initial Screening.

5.5. Criteria for Temporarily Delaying Enrolment, Randomization, or Administration of Study Intervention

Not applicable.

9. STATISTICAL CONSIDERATIONS

9.1. Statistical Hypotheses

No statistical hypotheses will be tested in this exploratory Phase 1/2a study.

9.2. Sample Size Determination

Approximately [redacted] participants are expected to be enrolled and treated in the study.

Approximately [redacted] participants will be enrolled and treated in Part 1, assuming [redacted] single participant cohorts and [redacted] cohorts of [redacted] participants and allowing for an additional [redacted] participants in the case of DLTs.

Approximately [redacted] participants will be enrolled and treated in Part 2, assuming up to [redacted] cohorts of [redacted] participants initially, and allowing for expansion of at least [redacted] cohorts with an additional [redacted] participants each (i.e., up to [redacted] participants per expanded cohort).

Note: "Enrolled" means a participant's, or their legally acceptable representative's, agreement to participate in a clinical study following completion of the informed consent process. Potential participants who are screened for the purpose of determining eligibility for the study, but do not participate in the study, are not considered enrolled, unless otherwise specified by the protocol.

The total number of participants screened will be reported.

9.3. Populations for Analyses

The analysis sets for this study are defined in Table 15.

Table 15: Populations for Analyses

Analysis Set	Description
Screened Analysis Set	All participants who sign the ICF
Safety Analysis Set (SAF)	All participants who receive at least 1 dose of investigational product.
SAD DLT Evaluable Set	All participants in the SAF who enroll into Part 1 and either experience a DLT or are evaluated for DLTs for at least [redacted] days
MAD Evaluable Set	All participants in the SAF who enroll into Part 2 and either experience a DLT or who receive at least [redacted] doses and are evaluated for at least [redacted] days from the start of the first dose
PK Analysis Set	All participants in the SAF who have had at least one blood sample providing evaluable PK data. The PK Analysis Set will be used for all PK data analyses. Note, participants may be removed from the estimation of certain PK parameters on an individual basis depending on the number of available blood samples. These participants will be identified at the time of analysis.
Immunogenicity Analysis Set (IAS)	All participants in the SAF who have had at least one blood sample providing evaluable ADA data. Participants may be removed from the IAS depending on the number of available blood samples for an individual participant. These participants will be identified at the time of analysis.
ATI Analysis Set	All participants in the SAF who stop ART and who have at least one blood sample providing evaluable pVL data after ART interruption. Participants may be removed from the ATI Analysis Set depending on the number of available blood samples for an individual participant. These participants will be identified at the time of analysis.

Abbreviations: ADA = anti-drug antibodies; ART = antiretroviral therapy; ATI = analytical therapy interruption; DLT = dose-limiting toxicity; IAS = immunogenicity analysis set; ICF = informed consent form; MAD = multiple ascending dose; PK = pharmacokinetic; pVL = plasma viral load; SAD = single ascending dose; SAF = safety analysis set.

Note: Participants who are treated but are not in their respective evaluable analysis set may be replaced.

9.4. Statistical Analyses

The SAP will be finalized prior to the database lock for the clinical study report (CSR) and will include a technical and detailed description of the statistical analyses. A summary of the planned statistical analyses of the most important endpoints including primary and secondary endpoints is presented below.

9.4.1. General considerations

The study data will be formally analyzed and reported in a CSR after all participants have completed the study and the database has been locked. Due to ongoing data entry and cleaning, summaries of DLT data in the CSR may not match the DLT data considered at the time of each dose escalation decision.

Data will generally be summarized using descriptive statistics. Categorical data will be presented as frequencies and percentages. For continuous data, the mean, standard deviation, median, minimum, and maximum will be presented. For the ATI phase of Part 2, the proportion of participants with pVL < 200 copies/mL after interruption of ART will be reported as a function of time.

Baseline is defined as the last assessment prior to the first dose of administered study treatment (e.g., pre-dose). Unless otherwise stated, missing data will simply be noted as missing on appropriate tables and listings.

Data will be presented separately by part (Part 1 - SAD and Part 2 - MAD). Within each part data will be summarized by cohort and overall.

Full details of data reporting will be outlined in the SAP.

9.4.2. Primary Endpoint(s)

Primary endpoints are listed in Section 3.

Incidence rates will be used to summaries primary endpoints relating to the incidence of AEs. The denominators for incidence rate calculations will be the Safety Analysis Set (SAF) for participants in the respective part of the study (Part 1 - SAD and Part 2 - MAD) except for summaries of DLTs, which will be based on the SAD DLT evaluable set and MAD DLT evaluable set. Numerators will generally be based on TEAEs, which are defined as an AE that occurs after the first dose of investigational product and within 28 days of the last dose of investigational product.

9.4.3. Secondary Endpoint(s)

Secondary endpoints are listed in Section 3. Details on the reporting of the secondary endpoints are listed in the following sections.

9.4.3.1. Pharmacokinetics

The PK Analysis Set will be used in all PK data analysis and PK summary statistics. The PK parameters that will be assessed are presented in Table 16.

Table 16: Pharmacokinetic Parameters

Parameter	Definition
AUC_{last}	The area under the curve (AUC) from time zero to the last measurable concentration sampling time (t_{last}) (mass × time × volume^{-1})
AUC_{inf}	The AUC from time zero to infinity (mass × time × volume^{-1})
C_{max}	The maximum (peak) observed plasma, blood, serum, or other body fluid drug concentration after single dose administration (mass × volume^{-1})
C_{ss}	The steady-state observed plasma, blood, serum, or other body fluid drug concentration after multiple dose administrations (mass × volume^{-1})
T_{max}	The time to reach maximum (peak) plasma, blood, serum, or other body fluid drug concentration after single dose administration (time)
$t_{½}$	The elimination half-life associated with the terminal slope (lz) of a semi logarithmic concentration-time curve (time). Use qualifier for other half-lives.
CL	The total body clearance of drug from the plasma (volume × time^{-1})

Descriptive statistics of all PK parameters for IMC-M113V will include arithmetic and geometric mean, median, standard deviation, and minimum and maximum. Zero concentrations will not be included in the geometric mean calculation. Since T_{max} is generally evaluated by a non-parametric method, median values and ranges will be given for this parameter. Missing concentration values will be reported as-is in data listings. Concentration values below lower limit of quantitation will be handled as zero in summary statistics and reported as is in data listings. Any missing PK parameter data will not be imputed.

Further analyses may be conducted using population PK approaches. In addition, a model-based approach may be used to explore the potential relationship between efficacy, safety, and/or biomarker endpoints and IMC-M113V concentration and/or exposure metrics. All analyses will be reported either in the CSR or a stand-alone report.

9.4.3.2. Immunogenicity

The IAS will be used in all immunogenicity analyses. The frequency and titer of anti-IMC-M113V anti-drug antibodies (ADA) at baseline and following treatment will be summarized.

The IMC-M113V concentration and immunogenicity relationship will be explored graphically and tabulated. The impact of ADA on PK, safety and efficacy will also be assessed as feasible to explore potential clinical impact of confirmed ADA on exposure, safety and efficacy of IMC-M113V. All analyses will be reported either in the CSR or a stand-alone report.

9.4.3.3. Pharmacodynamics

Lymphocyte count and cytokine/chemokine concentration data will be listed, summarized, and graphically displayed. Assessments at Screening, on-treatment, and change from baseline will be listed by participant and summarized (when sample size is sufficient) using descriptive statistics. The correlation between IMC-M113V dose level and changes in lymphocyte counts and cytokine concentrations will be explored.

9.4.3.4. Post-Treatment Control During ATI

The following parameters will be listed:

- The proportion of participants with pVL < 200 copies/mL 12 weeks after interruption of ART (W24)
- The proportion of participants resuming ART before W24
- The duration of post-treatment control (pVL < 200 copies/mL) after interruption of ART
- The duration of virological suppression (pVL < [redacted] copies/mL) after interruption of ART

The cumulative proportion of participants with pVL < 200 copies/mL over time will be graphically displayed and analyzed using Kaplan-Meier statistics.

9.4.4. Tertiary/Exploratory Endpoints

Exploratory endpoints will be evaluated according to a separate analysis plan and in a separate technical report to be included as an attachment to the CSR.

9.5. Interim Analyses

There is no formal interim analysis for this study.

Following the DLT observation period for each cohort of participants, data will be entered and evaluated for DLTs.

A trial monitoring table () will be used to guide decisions to stay, escalate, or de-escalate the dose given the observed number of DLTs among a given number of participants evaluable for DLT determinations.

These guidelines will be based on an mTPI-2 design, with modifications to prevent escalation prior to having DLT-evaluable participants within a given cohort (Guo et al. 2017). The target toxicity probability interval will be [redacted] %.

Safety will be continuously monitored by the Investigator, Sponsor's Medical Monitor and study personnel.

The Sponsor and Investigator(s) will conduct study data reviews as follows:

- Review of safety data for each SAD cohort in Part 1, to determine if a dose escalation can be implemented.
- Review of safety data in Part 1 to assess the need for addition of corticosteroids as part of the mandatory CRS premedication.
- Review of safety and PD biomarker data in Part 1, to determine if a cohort meets PAD criteria to initiate the first MAD cohort in Part 2.
- Review of cumulative SAD and MAD safety, PK and PD data up to Day 29, at minimum, or up to Day 84 together with pVL data during weeks 12-18 of the ATI phase, if available, to determine if dose escalation to a subsequent MAD cohort can be initiated.

Safety data will also be summarized and evaluated for DMC meetings, as described in Section 9.6.

9.6. Data Monitoring Committee

Two levels of data oversight will occur in this study:

- An SST will be convened, comprising participating Investigators, the Sponsor Medical Monitor, and statistician. The SST will be responsible for dose decision-making, including escalations and modifications for reasons of safety.

For each cohort, dose escalation decisions will be based on the SST's review of all relevant data available, from all dose levels evaluated in the ongoing study, cumulative including safety (AEs, DLTs, laboratory data, vital signs, and ECG data), PK, and PD data. These data will be discussed, and dose recommendations will be made during a dose escalation teleconference. All decisions will be documented and shared with the Investigators and site personnel. The SST have the following responsibilities:

- Review of safety data for each SAD cohort in Part 1, to determine if a dose escalation can be implemented
- Review of safety data in Part 1 to assess the need for the addition of corticosteroids as part of the mandatory CRS premedication

- Review of safety and PD biomarker activity data in Part 1, to determine if a cohort meets PAD criteria to initiate the first MAD cohort in Part 2
- Recommend whether to remove requirement for extended monitoring for a given dose during Part 2 based on emerging data from Part 1 or Part 2. May also recommend what additional safeguards are needed when extended monitoring is removed for a given dose.
- Review of cumulative SAD and MAD safety, PK, and PD data in Part 2, together with pVL data during weeks 12-18 of the ATI phase, if available, to determine if dose escalation to a subsequent MAD cohort can be initiated
- Determination of whether additional participants should be added to a given dose cohort provided it is permitted by the mTPI-2 algorithm

An independent DMC will be convened. The DMC will be comprised of 3 members, who will be independent of the Sponsor and Investigators, and will operate in accordance with a prespecified charter and make recommendations to the Sponsor. At a minimum, the DMC will convene at the following times:

- When safety data from the Day 29 visit are available for all the cohorts in Part 1.
- After completion of Part 2.

Additional ad-hoc DMC meeting(s) may be scheduled on request from the Sponsor or the DMC. Further details will be provided in the DMC charter.
