# Supplementary material for: Safety and biologic activity of a bispecific T cell receptor targeting HIV Gag in males living with HIV: a first-in-human trial
Source: Nat Commun. 2026 Jan 31;17:2207. doi: 10.1038/s41467-026-68833-2 (PMC12963441; doi:10.1038/s41467-026-68833-2)
Supplement: Supplementary file 3 — Reporting Summary [file 41467_2026_68833_MOESM3_ESM.pdf]

nature portfolio

Corresponding author(s): Linos Vandekerckhove

Last updated by author(s): January 9, 2026

Reporting Summary

Nature Portfolio wishes to improve the reproducibility of the work that we publish. This form provides structure for consistency and transparency in reporting. For further information on Nature Portfolio policies, see our Editorial Policies and the Editorial Policy Checklist.

Please do not complete any field with "not applicable" or n/a. Refer to the help text for what text to use if an item is not relevant to your study.
For final submission: please carefully check your responses for accuracy; you will not be able to make changes later.

Statistics

For all statistical analyses, confirm that the following items are present in the figure legend, table legend, main text, or Methods section.

n/a	Confirmed	
☐	☒	The exact sample size (*n*) for each experimental group/condition, given as a discrete number and unit of measurement
☐	☒	A statement on whether measurements were taken from distinct samples or whether the same sample was measured repeatedly
☐	☒	The statistical test(s) used AND whether they are one- or two-sided<br>*Only common tests should be described solely by name; describe more complex techniques in the Methods section.*
☒	☐	A description of all covariates tested
☐	☒	A description of any assumptions or corrections, such as tests of normality and adjustment for multiple comparisons
☐	☒	A full description of the statistical parameters including central tendency (e.g. means) or other basic estimates (e.g. regression coefficient) AND variation (e.g. standard deviation) or associated estimates of uncertainty (e.g. confidence intervals)
☐	☒	For null hypothesis testing, the test statistic (e.g. *F*, *t*, *r*) with confidence intervals, effect sizes, degrees of freedom and *P* value noted<br>*Give P values as exact values whenever suitable.*
☒	☐	For Bayesian analysis, information on the choice of priors and Markov chain Monte Carlo settings
☒	☐	For hierarchical and complex designs, identification of the appropriate level for tests and full reporting of outcomes
☐	☒	Estimates of effect sizes (e.g. Cohen's *d*, Pearson's *r*), indicating how they were calculated

Our web collection on statistics for biologists contains articles on many of the points above.

Software and code

Policy information about availability of computer code

Data collection	Not applicable
Data analysis	Statistical analyses were performed using GraphPad prism version 10 or later.<br><br>Quantification of cell associated HIV RNA: Droplets were analysed on a QX200 Droplet Reader (Bio-Rad) using QXManager software (Bio-Rad, version 1.2)<br><br>Quantification of intact proviral DNA was performed using QIAcuity Software Suite 2.5.0.1 (Qiagen)<br><br>Sequencing of Gag77-85 from HIV CA-RNA:<br>For sequence analyses, Fastq files were processed as follows: Quality and adaptor trimming was performed using Trim Galore (v0.6.2) with default parameters, specifying the amplification primers and Nextera adaptors. FastQC was used to assess read quality. Mapping was carried out against the HIV-1 HXB2 reference annotation (obtained from NCBI November 2022; ASM310297v1 / GCA_003102975.1) using BWA INDEX and BWA MEM (v0.7.17) with PCR duplicates removed. MultiQC was used to assess mapping statistics. iVar variants (v1.3.1) was used to call variants from the aligned BAM files and iVar consensus (v1.3.1) used to generate consensus sequences. SAMtools Depth (v1.3) was used to compute the alignment depth along the HIV-1 genome. Sequence plotting and visualisation was performed in R using the packages muscle (v3.38.0), seqinr (v4.2.16), ggplot2 (3.3.6) and ggmsa (v1.2.3). Variants obtained in the region of interest (HIV Gag77-85) at a frequency of > 3% were reported.

For manuscripts utilizing custom algorithms or software that are central to the research but not yet described in published literature, software must be made available to editors and reviewers. We strongly encourage code deposition in a community repository (e.g. GitHub). See the Nature Portfolio guidelines for submitting code & software for further information.

Data

Policy information about availability of data

All manuscripts must include a data availability statement. This statement should provide the following information, where applicable:

- Accession codes, unique identifiers, or web links for publicly available datasets
- A description of any restrictions on data availability
- For clinical datasets or third party data, please ensure that the statement adheres to our policy

The following data availability statement is included in the manuscript: Key elements of the IMC-M113V-103 study protocol are available at the European Union Clinical Trials Register (EudraCT 2021-002008-11). A redacted version of the IMC-M113V-103 study protocol is included in the Supplementary Information file and at CTIS - Clinical Trials in the European Union (https://euclinicaltrials.eu). Source data are provided with this paper. Cell associated HIV *gag* RNA sequences generated in this study have been deposited in the BioSample database under BioProject PRJNA1372654 (https://www.ncbi.nlm.nih.gov/bioproject/?term=PRJNA1372654; https://trace.ncbi.nlm.nih.gov/Traces/study/?acc=SRP650654&o=acc_s%3Aa).

Research involving human participants, their data, or biological material

Policy information about studies with human participants or human data. See also policy information about sex, gender (identity/presentation), and sexual orientation and race, ethnicity and racism.

Reporting on sex and gender	Demographic information included in the manuscript. Sex and gender analyses not applicable as this is a first in human single arm study with limited sample size (n=12).
Reporting on race, ethnicity, or other socially relevant groupings	Demographic information included in the manuscript but not applicable for analyses as this is a first in human single arm study with limited sample size (n=12).
Population characteristics	Participants were people with HIV aged 18-65 years, with a positive test for HLA-A*02:01 a assessed by a central laboratory assay, treatment with ART for a minimum of 1 year and maximum 7 years at the time of planned first dose, with pVL <50 copies/ml, CD4+ T cell count >500 cells/µl and CD4+ T cell nadir >200 cells/µl. Participants were excluded if they were a known HIV controller (pVL <2000 copies/ml in the absence of ART for at least 12 months) or had initiated ART within 12 weeks of a confirmed diagnosis of primary HIV infection, or had active co-infection with hepatitis B or C virus. Baseline characteristics are provided in manuscript (Table 1).
Recruitment	Potentially eligible participants were prescreened for the HLA-A*02:01 allele. HLA-A*02:01-positive participants were then invited to screen for eligibility. Recruitment was undertaken at hospital sites in the United Kingdom, Belgium and Spain.
Ethics oversight	Ethical approval was obtained from East of England – Cambridge East Research Ethics Committee, UK (21/EE/0242), University of Gent Medical Ethics Committee (BC-11136), Belgium and the Drug Research Ethics Committee of the Hospital Universitario Germans Trias y Pujol, Spain.

Note that full information on the approval of the study protocol must also be provided in the manuscript.

Field-specific reporting

Please select the one below that is the best fit for your research. If you are not sure, read the appropriate sections before making your selection.

☒ Life sciences ☐ Behavioural & social sciences ☐ Ecological, evolutionary & environmental sciences

For a reference copy of the document with all sections, see nature.com/documents/nr-reporting-summary-flat.pdf

Life sciences study design

All studies must disclose on these points even when the disclosure is negative.

Sample size	No formal sample size calculation was performed as this is a first in human single arm dose finding study.
Data exclusions	Quantification of cell associated HIV RNA: Day 8 time points for P4, P5, and P8 excluded due to poor yields/viability of CD4 T cells isolated from PBMCs and poor yields of RNA extracted from the cells.
Replication	Not applicable
Randomization	Randomization not performed because this is a single arm study
Blinding	Blinding not performed because this is a single arm open label study

Reporting for specific materials, systems and methods

We require information from authors about some types of materials, experimental systems and methods used in many studies. Here, indicate whether each material, system or method listed is relevant to your study. If you are not sure if a list item applies to your research, read the appropriate section before selecting a response.

Materials & experimental systems

n/a	Involved in the study
☐	☒ Antibodies
☐	☒ Eukaryotic cell lines
☒	☐ Palaeontology and archaeology
☒	☐ Animals and other organisms
☐	☒ Clinical data
☒	☐ Dual use research of concern
☒	☐ Plants

Methods

n/a	Involved in the study
☒	☐ ChIP-seq
☐	☒ Flow cytometry
☒	☐ MRI-based neuroimaging

Antibodies

Antibodies used

HIV Gag p24 intracellular staining:
CD3 APC*Cy7 HIT3a 300318 BioLegend
CD8 APC SK1 344722 BioLegend
P24 FITC KC57 6604665 Beckman Coulter
CCR7 BUV395 2-L1-A 749655 BD Biosciences
CD25 BUV563 2A3 612918 BD Biosciences
CD28 BUV615 L293 751270 BD Biosciences
CD39 BUV661 TU66 749967 BD Biosciences
CD8 BUV805 SK1 612889 BD Biosciences
CD45RO BV570 UCHL1 304226 Biolegend
CD3 APC-Vio770 REA613 130-113-136 Miltenyi
CD4 BUV496 SK3 612936 BD Biosciences
CD95 BUV737 DX2 612790 BD Biosciences
IL-2 BV421 MQ1-17H12 564164 BD Biosciences
CD154 (CD40L) BV480 TRAP1 746337 BD Biosciences
IL17A BV605 BL168 512326 Biolegend
Ki-67 BV650 B56 563757 BD Biosciences
CD69 BV711 FN50 563836 BD Biosciences
TNFa BV750 Mab11 566359 BD Biosciences
Granzyme B FITC GB11 560211 BD Biosciences
IL-6 BB630 MQ2-13A5 Custom BD Biosciences
IL-13 BB660 JES10-5A2 Custom BD Biosciences
IL-4 BB660 8D4-8 Custom BD Biosciences
IFNg BB700 B27 566394 BD Biosciences
Perforin AX594 B-D48 NBP3-14581AF594 Novus Biologics
FoxP3 PE.Cy5.5 PCH101 35-4776-42 Thermo Fisher Scientific

Validation

All the antibodies above were commercially sourced, supplier and catalogue numbers are provided above and in Supplementary table 2.

Eukaryotic cell lines

Policy information about cell lines and Sex and Gender in Research

Cell line source(s)

T2 cells (#CRL-1992) were obtained from American Type Culture Collection (ATCC; Manassas, VA).
C8166 cells (#88051601) were obtained from European Collection of Authenticated Cell Cultures (ECACC, UK).
Primary Human Bronchial Epithelial Cells (HBEpiC) were obtained from Promocell (#C-12640) (2 female, 1 male lots)
Induced Pluripotent Stem Cells (iPSC) derived astrocytes (Astro1g) were obtained from Cellular Dynamics (#ASC-100-020-001-PT) (1 female lot)
Induced Pluripotent Stem Cells (iPSC) derived cardiomyocytes were obtained from Cellular Dynamics (#C1006) (2 female, 1 male lots)

Authentication

Cell lines were authenticated by short tandem repeat (STR) analysis

Mycoplasma contamination

All cell lines tested negative for Mycoplasma contamination

Commonly misidentified lines
(See ICLAC register)

Name any commonly misidentified cell lines used in the study and provide a rationale for their use.

Clinical data

Policy information about clinical studies

All manuscripts should comply with the ICMJE guidelines for publication of clinical research and a completed CONSORT checklist must be included with all submissions.

Clinical trial registration

EudraCT 2021-002008-11

Study protocol

A redacted version of the IMC-M113V-103 study protocol is included in the Supplementary Information file and at CTIS - Clinical Trials in the European Union (https://euclinicaltrials.eu).

Data collection

First patient first visit 12th April 2022
Last patient last visit - 12th January 2023

Outcomes

The predefined outcomes and methods of assessment are described in the protocol. Primary outcome (Safety and tolerability of intervention) assessed by:
- Incidence and severity of treatment-emergent adverse events (TEAEs)
- Incidence of dose-limiting toxicities (DLTs)
- Changes in safety laboratory parameters, vital signs, and electrocardiogram (QTcF)
- Incidence of serious adverse events (SAEs) and AEs leading to treatment interruption, dose reduction, or discontinuation through 28 days after the last infusion of study treatment

Secondary outcomes were as follows:
- Pharmacokinetics profile: Parameters (eg, AUC, Cmax, Tmax, t1/2) assessed at multiple time points from baseline up to 72 hours post-dose
- Incidence of anti-IMC-M113V antibody formation following single infusion
- Pharmacodynamic changes in the systemic immune response in relation to treatment with IMC-M113V: Change in serum cytokines/chemokines and peripheral blood lymphocyte counts

Plants

Seed stocks

Report on the source of all seed stocks or other plant material used. If applicable, state the seed stock centre and catalogue number. If plant specimens were collected from the field, describe the collection location, date and sampling procedures.

Novel plant genotypes

Describe the methods by which all novel plant genotypes were produced. This includes those generated by transgenic approaches, gene editing, chemical/radiation-based mutagenesis and hybridization. For transgenic lines, describe the transformation method, the number of independent lines analyzed and the generation upon which experiments were performed. For gene-edited lines, describe the editor used, the endogenous sequence targeted for editing, the targeting guide RNA sequence (if applicable) and how the editor was applied.

Authentication

Describe any authentication procedures for each seed stock used or novel genotype generated. Describe any experiments used to assess the effect of a mutation and, where applicable, how potential secondary effects (e.g. second site T-DNA insertions, mosiacism, off-target gene editing) were examined.

Flow Cytometry

Plots

Confirm that:

- [x] The axis labels state the marker and fluorochrome used (e.g. CD4-FITC).
- [x] The axis scales are clearly visible. Include numbers along axes only for bottom left plot of group (a 'group' is an analysis of identical markers).
- [x] All plots are contour plots with outliers or pseudocolor plots.
- [x] A numerical value for number of cells or percentage (with statistics) is provided.

Methodology

Sample preparation

Intracellular Gag p24 staining was used to quantify HIV infected C8166 cells (ECACC #88051601) using a previously published method (Yang, H. et al. J Immunol Methods 391, 174–178 (2013)
T cell activation and effector function were assessed in PBMC from study participants as described in Methods.

Instrument

Intracellular Gag p24 staining - MACSQuant X (Miltenyi)
T cell activation and effector function - ID7000 spectral analyser (Sony)

Software

Intracellular Gag p24 staining - FlowJo (v10)
T cell activation and effector function - Omiq (OMIQ.ai)

Cell population abundance

Not applicable

Gating strategy

Intracellular Gag p24 staining:
Singlets - Live cells - Lymphocytes - CD3+ CD8- to distinguish target cells from effector cells - p24+ve cells to identify infected

cells
T cell activation and effector function:
Singlets - Live cells - Lymphocytes - CD3+ - CD4+ or CD8+ - individual marker (cytokine or other effector molecule)

☒ Tick this box to confirm that a figure exemplifying the gating strategy is provided in the Supplementary Information.

This checklist template is licensed under a Creative Commons Attribution 4.0 International License, which permits use, sharing, adaptation, distribution and reproduction in any medium or format, as long as you give appropriate credit to the original author(s) and the source, provide a link to the Creative Commons license, and indicate if changes were made. The images or other third party material in this article are included in the article's Creative Commons license, unless indicated otherwise in a credit line to the material. If material is not included in the article's Creative Commons license and your intended use is not permitted by statutory regulation or exceeds the permitted use, you will need to obtain permission directly from the copyright holder. To view a copy of this license, visit http://creativecommons.org/licenses/by/4.0/
